# Prey Acceptability and Preference of *Oenopia conglobata* (Coleoptera: Coccinellidae), a Candidate for Biological Control in Urban Green Areas

**DOI:** 10.3390/insects9010007

**Published:** 2018-01-12

**Authors:** Belén Lumbierres, Filipe Madeira, Xavier Pons

**Affiliations:** Universitat de Lleida. Department of Crop and Forest Sciences. Agrotecnio Center. Av. Rovira Roure 191, 25198 Lleida, Spain; bel.lumbierres@gmail.com (B.L.); fmadeira@pvcf.udl.cat (F.M.)

**Keywords:** cocinellids, predator, aphids, Hemiptera, Aphididae, Psyllidae, biological control agent

## Abstract

*Oenopia conglobata* is one of the most common ladybird species in urban green areas of the Mediterranean region. We have obtained data about its prey acceptability and prey preferences. In a laboratory experiment, we investigated the acceptability of seven aphid and one psyllid species as prey for this coccinellid: the aphids *Chaitophorus populeti, Aphis gossypii*, *Aphis craccivora* *Monelliopsis caryae*, *Eucallipterus tiliae*, *Aphis nerii* (on white poplar, pomegranate, false acacia, black walnut, lime, and oleander, respectively), and the psyllid *Acizzia* *jamatonica* on Persian silk tree. These species are abundant in urban green areas in the Mediterranean region. In addition, we tested the acceptability of *Rhopalosiphum padi* on barley, an aphid species easily reared in the laboratory. We also tested preferences of the predator in cafeteria experiments with three aphid species and one aphid and the psyllid. Adults and larvae of the coccinellid accepted all of the preys offered, except *A. nerii*, with a clear preference for *M. caryae*. The predator also showed preference for *M. caryae* when it was offered in a cafeteria experiment with other aphid species or with the psyllid. The aphid *R. padi* obtained a good acceptability and could be used for rearing *O. conglobata* in the laboratory.

## 1. Introduction

The ladybird *Oenopia conglobata* (Coleopera: Coccinellidae) is a common species on trees and shrubs in urban green areas in several countries of Central Europe and the Mediterranean basin [1,2,3,4], but it has also been cited on herbaceous plants in South Europe and Asia [1,5,6]. However, information about the ecology of this predator is scarce. It has been recorded to mainly prey on aphids (Hemiptera: Aphididae) and psyllids (Hemiptera: Psyllidae) [7,8].

In the Northeast of the Iberian Peninsula, aphids, and lately psyllids are some of the most frequent and annoying pests of urban green areas. They damage plants by sap sucking and excrete honeydew that is deposited on the vegetation, pavements, cars, and urban furniture [9]. *Oenopia conglobata* is one of the most common ladybird species occurring in urban landscapes, preying mainly on aphids [2,9], and with lesser extent on psyllids, mites and mealybugs [10].

The success of the predator for pest control depends in part upon the willingness to consume the target prey. This study aims to obtain data about the prey acceptability of *O. conglobata*. Knowledge about the range of suitable prey and feeding preferences may provide initial information about the potential of this ladybird as biological control agent in urban green areas.

## 2. Materials and Methods

The experiments were conducted in the laboratory of Entomology of the department of Crop and Forest Sciences of the University of Lleida. A colony of *O. conglobata* was reared and maintained in climate chamber at 25 ± 2 °C, 60% RH (relative humidity) and a 16:8 (light:dark) photoperiod on eggs of *Ephestia kuehniella* Zellner (Lepidoptera: Pyralidae) and pollen. The origin of the colony was overwintering adults collected under bark of plane trees (*Platanus* sp.) in different locations around the city of Lleida. The colony was yearly renewed by adding wild adults. Newly emerged (<24 h) male and female adults and 3rd instar larvae (L3) from this colony were tested. Adults were starved for 24 h and larvae for 12 h before starting the experiments.

Among the aphid species, we selected some aphids common on trees in urban green areas of the NE Iberian Peninsula and were chosen in function of field surveillance evidence: *Chaitophorus populeti* Panzer on white poplar (*Populus alba* L.), *Monelliopsis caryae* Monell on black walnut (*Juglans nigra* L.), *Eucallipterus tiliae* L. on lime (*Tilia* sp.), *Aphis gossypii* Glover on pomegranate (*Punica granatum* L.), and *Aphis craccivora* Koch on false acacia (*Robinia pseudoacacia* L.). We also selected *Aphis nerii* Boyer de Fonscolombe on oleander (*Nerium oleander* L.), because it is reported as an aphid species disliked by coccinellids and we want to verify this for *O. conglobata*. In addition, we included *Rhopalosiphum padi* L. on barley (*Hordeum vulgare* L.) in the experiment, an aphid species easily reared in the laboratory but not reported as a prey for *O. conglobata*. The psyllid *Acizzia jamatonica* Kuwayama on Persian silk tree (*Albizia julibrissin* Durazz.) was also tested.

### 2.1. Non-Choice Prey Acceptability

Test arenas consisted of a 9 cm diameter plastic Petri dish equipped with one clean leaf of the host tree infested with 20 individuals of the associated aphid or psyllid. Fourth instar nymphs of the aphids and the psyllid were used, in order to avoid flight and aphid offspring. One adult or one L3 instar larva of *O. conglobata* was released into each arena. Arenas were sealed with parafilm to keep the prey from escaping. No prey individuals were replaced. The number of prey individuals consumed at 1, 2, 4, and 8 h after coccinellid release were recorded. The sex of the adult coccinellids was determined at the end of the experiment. The experiment was conducted at 25 ± 2 °C and 60% RH. Three runs of the experiment were conducted and the total number of replications for each combination of prey species and predator life stage (adults and larvae) ranged from 30 to 34. Each individual of *O. conglobata* was only used once in the experiment.

### 2.2. Choice (Cafeteria) Experiments

We also tested preferences of the predator in two choice experiments between: a) three aphid species commonly preyed on by *O. conglobata* in urban green areas [9]: *M. caryae*, *C. populeti,* and *E. tiliae*; and, b) one aphid species (*M. caryae*) and the psyllid *A. jamatonica*.

The experimental set-up in both experiments was similar to the one described above, but included 20 individuals on one leaf of the respective host plants of each of the three aphid species or of the aphid and psyllid per arena. Experimental conditions were the same as in the non-choice experiment and a 16:8 (light:dark) photoperiod. The number of prey species consumed by adults and larvae of *O. conglobata* at 1, 2, 4, 8, and 24 h in each arena was recorded. The prey species of each arena that was attacked first and the time that had elapsed was also recorded. Three runs of the experiment with 10 replications each were performed. Each individual of *O. conglobata* was only used once in the experiment.

### 2.3. Data Analysis

#### 2.3.1. Non-Choice Prey Acceptability

Differences between the cumulated prey consumed at 1, 2, 4, and 8 h were analyzed by ANOVA through the General Linear Model (GLM) procedure after log (*x* + 1) transformation where *x* was the cumulated number of prey eaten. The prey consumption rate at 8 h for each prey species and arena was calculated as ((number of aphids consumed/(20 − *d*)) × 100), where *d* was the number of aphid deaths by causes other than predation. After transformation to (arcsin *x*^1/2^), where *x* was the consumption rate, ANOVA was performed through the GLM procedure. The variation factors were always the run, the coccinellid life stage (adult or larva), the prey species, and the interaction between prey species and coccinellid life stage. When differences between prey species were found, means were compared by the Tukey’s test.

#### 2.3.2. Choice (Cafeteria) Experiments

To measure the prey preference of each isolated individual predator, we used the index of Rodgers for cafeteria experiments [11]. For that, the area under the cumulative consumption curve versus time (1–24 h) for each available prey was calculated and then standardized according to *Ri = Ai/max (Ai)*, where *Ri* is Rodgers’s index of preference for species *i*, *Ai* is the area under the cumulative consumption curve for species *i*, and *max (Ai)* is the largest value of *Ai*. For each individual predator the most preferred prey was given a preference score of 1.0 and the least preferred the lowest score. Rodgers’s preference indexes were compared by ANOVA through GLM procedure after log (*x* + 1) transformation. The run, the coccinellid stage (adult or larva), the prey species, and the interaction prey species and coccinellid stage were the variation factors. Differences between adult males and females were also analyzed. When differences between preys were found, means were compared by the Tukey’s test.

The times at which each prey species was first attacked in the two cafeteria experiments and the time that had elapsed until the first attack were analyzed through a Fisher’s test and GLM, respectively.

Data were analyzed using the SAS statistical package version 9.1 [12].

## 3. Results

### 3.1. Non-Choice Prey Acceptability

Eight hours after the release of *O. conglobata* into the arenas, the consumption rate of the coccinellid significantly differed depending on the prey species (*F* = 227.83; *P* < 0.0001; *df* = 7, 480), the coccinellid life stage (*F* = 145.36; *P* < 0.0001; *df* = 1, 480), and the interaction of the two factors (*F* = 6.80; *P* < 0.0001; *df* = 7, 480). Therefore, adults and larvae were analyzed separately.

**Figure 1:**
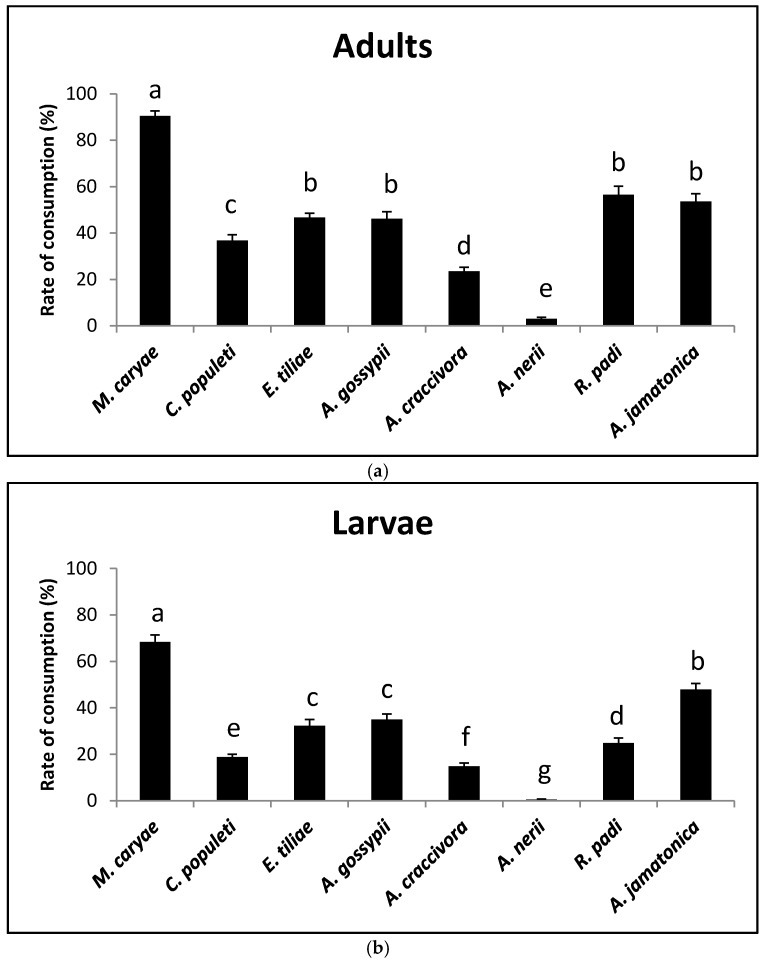
Prey consumption rate of adults (**a**) and larvae (**b**) of *O. conglobata* after 8 h with different aphid and psyllid prey species in the non-choice experiment. For each coccinellid life stage, columns with different letters above are significantly different.

There were differences in the consumption rates of adults on the different prey species (*F* = 133.98; *P* < 0.0001; *df* = 7, 231). Both adult genders of *O. conglobata* accepted all of the prey offered, but females showed a higher consumption rate than males (*F* = 35.86; *P* < 0.0001; *df* = 1, 231). There was no significant difference in the interaction prey and sex (*F* = 1.52; *P* = 0.1625; *df* = 7, 231. The acceptability of *M. caryae* was the highest (90.5%) and that of *A. nerii* was the lowest (3%) (Figure 1a). The psyllid *A. jamatonica* and the aphid *R.padi* also showed a high acceptability (more than 50%), which did not statistically differ from that of *E. tiliae* and *A. gossypii* that were lower. The consumption rate of *C. populeti* was 37%, but did not statistically differ from that of *E.tiliae and A. gossypii*. *Aphis craccivora* showed a low rate of acceptability (around 20%).

Larvae showed lower consumption rates than adults. As with adult coccinellids there were significant differences in the acceptability of the prey species (*F* = 117.42; *P* < 0.0001; *df* = 7, 239). The most accepted prey was again the aphid *M. caryae* (68%). *A. nerii* was practically unaccepted (Figure 1b). Significant differences were also found between the consumption rate of *A. jamatonica* (48%) and the remaining aphid species (15% to 35%). *Aphis craccivora* showed a low acceptability again.

The number of individuals of each prey consumed by adults and larvae at 1, 2, 4, and 8 h after *O. conglobata* was released into the arena can be seen in Table 1 and shows that after 1 h *M. caryae* was already the most consumed prey. This occurred during the entire experiment.

### 3.2. Choice (Cafeteria) Experiment

#### 3.2.1. Preference among Aphid Species

*Chaitophorus populeti* was the aphid that was attacked first in 13 out of 31 arenas, followed by *M. caryae* and *E. tiliae* in 9 and 8 arenas, respectively. There were no differences between prey species in this first attack (*P* = 0.4581). The time elapsed until the first attack was very variable and ranged from 30 to 1200 s. There were neither differences between the prey species (*F* = 1.22; *F* = 0.3031; *df* = 2, 52), coccinellid life stage (*F* = 1.63; *F* = 0.2071; *df* = 1, 52) nor the interaction of prey species and coccinellid stage (*F* = 0.49; *F* = 0.6127; *df* = 2, 52).

**Figure 2:**
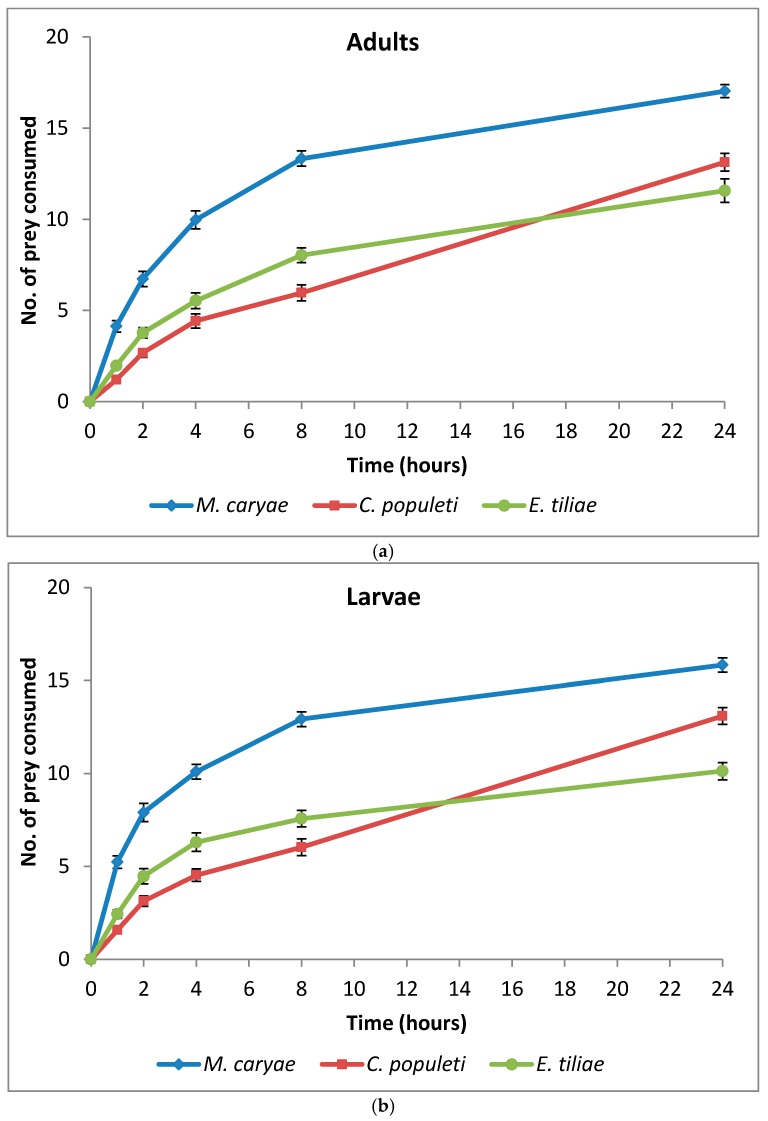
Cumulative number (mean ± s.e.) of aphids eaten by adults (**a**) and larvae (**b**) of *O. conglobata* after 24 h when the three aphids were offered simultaneously. The number of replications for adults and larvae were *n* = 30 each.

The number of *M. caryae*, *E. tiliae* and *C. populeti* eaten by adults and larvae of *O. conglobata* are shown in Figure 2.

After 24 h, adults and larvae of the coccinellid had not consumed all of the prey offered in the arena but the number of aphids eaten steady increased until the end of the experiment. *Oenopia conglobata* had a preference for some of the aphid species included in the experiment (*F* = 124.44; *P* < 0.0001; *df* = 2, 172), but there were no differences between the preference of adults and larvae (*F* = 0.59; *P* = 0.4450; *df* = 1, 172) and no interaction prey and coccinellid life stage occurred (*F* = 0.28; *P* = 0.7591; *df* = 2, 172). No significant differences between adult sex (*F* = 2.14; *P* = 0.1473; *df*= 1, 82) and the interaction sex and prey (*F* = 0.62; *P* = 0.5427; *df* = 2, 82) were found either. The aphid *M. caryae* was the preferred prey of adults and larvae of *O. conglobata* (Table 2) and it was consumed in a high proportion (Figure 2). On the other hand, there were no differences between the preference of the coccinellid for *C. populeti* or *E. tiliae* (Table 2).

#### 3.2.2. Preference between Aphid and Psyllid

The psyllid was the first prey attacked in 16 arenas, whereas the aphid was the first to be attacked in 15 arenas. Consequently, no differences between prey were detected (*P* = 1.0). The time elapsed until the first attack of the aphid was also very variable and ranged from 5 to 1200 s, similar to the choice experiment with the three aphids. There were no significant differences between prey species (*F* = 1.11; *P* = 0.2973; *df* = 1, 61), the coccinellid life stage (*F* = 0.23; *P* = 0.6271; *df* = 1, 61), or the interaction between these two factors (*F* = 0.12; *P* = 0.7323; *df* = 1, 61).

**Figure 3:**
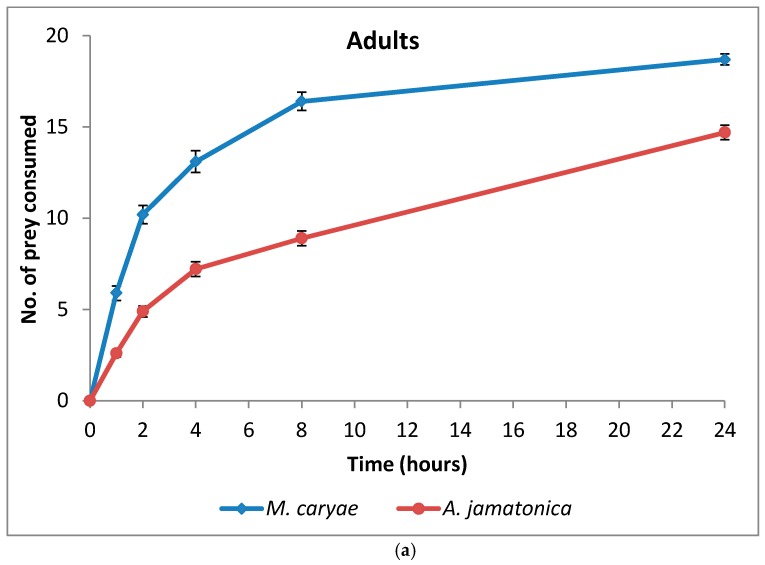

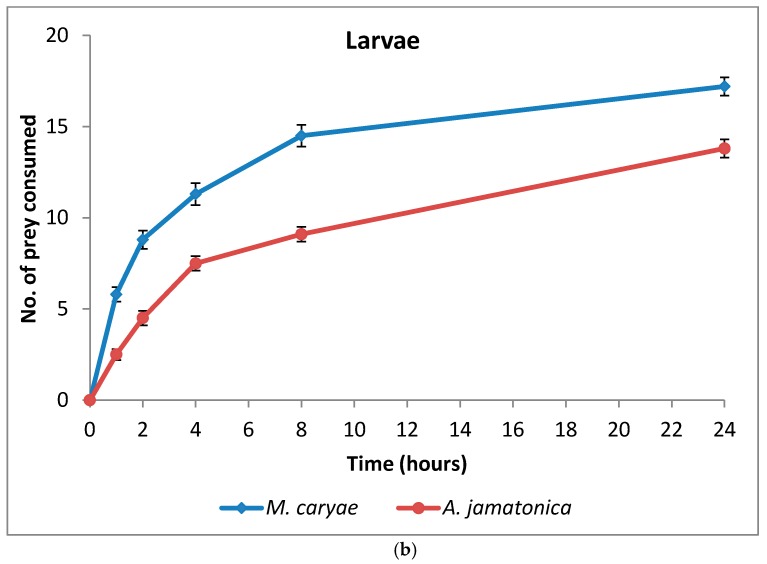
Cumulative number (mean ± s.e) of prey eaten by adults (**a**) and larvae (**b**) of *O. conglobata* after 24 h when one aphid and one psyllid were offered simultaneously. The number of replications for adults and larvae were *n* = 31 each.

The number of *M. caryae* and *A. jamatonica* eaten by adults and larvae of *O. conglobata* are shown in Figure 3. After 24 h, adults and larvae of the coccinellid had not consumed all aphids or psyllids offered in the arena, but the number of aphids eaten increased fast until 8 h (82%). Afterwards the consumption rate increased slowly as occurred in the experiment of aphid preference. *Oenopia conglobata* had a preference for the aphid (*F* = 203.52; *P* < 0.0001; *df* = 1, 118), but there were not differences between the preferences of adults and larvae (*F* = 0.43; *P* = 0.5153; *df* = 1, 172) (Table 3) and no interaction prey and coccinellid life stage occurred (*F* = 2.60; *P* = 0.1097; *df* = 1, 172). Significant differences between adult sex (*F* = 0.00; *P* = 0.9592; *df* = 1, 56) and the interaction sex and prey (*F* = 1.88; *P* = 0.1756; *df* = 1, 56) were not found either.

## 4. Discussion

In the Mediterranean regions the aphids *M. caryae*, *C. populeti*, *E. tiliae*, *A. gossypii*, *A. craccivora,* and *A. nerii,* and the psyllid *A. jamatonica* are common pests on trees and shrubs of urban green areas and their period of activity coincides with that of the coccinellid *O. conglobata* [9]. Therefore, all of these pests may be considered to be a potential prey for the coccinellid. The other aphid, *R. padi*, is an holocyclic species that uses the bird-cherry *Prunus padus* L. as primary and Poaceae as secondary hosts [13]. In the Iberian Peninsula and in most of the Mediterranean area, populations of *R. padi* are mainly anholocyclic and recorded as an important pest of cereals [14]. Although some overwintering populations have been recorded on ornamental *P. padus* in Lleida [15], populations abandon these trees very early in spring and do not coincide with the activity of *O. conglobata*. Therefore, *R. padi* cannot be considered a potential prey of the coccinellid in open air conditions.

In the non-choice experiments, the mean consumption rate never reached 100% at 8 h after the release of the predator into the arena. This points out that the number of aphids included in the arena was high enough to discriminate between prey acceptability. Sadegi et al. [16] and Mojib Hagh ghadam [17] reported that within 24 h adults and L3 larvae of *O. conglobata* are able to eat a range of 37–45 and 36–40 aphids, respectively. However, in our experiment, adults consumed more aphids than larvae. This could be due to the higher mobility of the adults, facilitating aphid encounters. Yaşar and Özger [18] also reported that adults of *O. conglobata* show a higher voracity than larvae.

The results that are presented in this study show that *O. conglobata* is able to accept five of the six aphid species offered, as well as the psyllid *A. jamatonica*. However, significant differences between the prey species acceptability suggest that not all of them are equally suitable. The inclusion of aphids on their host plants into the arenas could have contributed to this because host plants may mediate the attack response by olfactory or visual stimuli [19,20]. For example, *M. caryae* showed the highest acceptability for both adults and larvae of *O. conglobata*. Both coccinellid stages also showed a higher acceptability of *A. jamatonica* than for the other four aphid species. *Oenopia conglobata* has been reported as a predator of psyllids in Europe [7] and the Middle East [8]. Furthermore, *R. padi* showed acceptability similar to *A. jamatonica* for adults and no different to that of other aphids for larvae. The ease of maintaining *R. padi* populations in the laboratory on cereal plants suggests a great potential of this aphid as a prey in more detailed studies on the fitness and functional responses of *O. conglobata*.

As expected, *A. nerii* was almost unaccepted. This aphid, feeding on oleander, has been reported as toxic for some ladybirds like *Coccinella septempunctata* L., *Adalia bipunctata* L., *Propylea quatuordecimpunctata* L., and *Coccinella undecimpunctata* L., but not for *Hippodamia variegata* (Goeze) [21]. However, toxic aphid species, such as *Aphis sambuci* L. and *Megoura viciae* Buckton can be accepted and consumed by some coccinellids in laboratory experiments contrary to observations in the field [22,23]. Our result is a verification of the presumable toxicity of *A. nerii* for *O. conglobata*, enhanced by the fact that we offered the aphids on an oleander leaf in the arenas. In fact, we have never recorded *O. conglobata* on oleanders; meanwhile, *H. variegata* is the dominant ladybird in *A. nerii* colonies.

When adults and larvae of *O. conglobata* were simultaneously presented with a choice of three aphids, both life stages showed a clear preference for *M. caryae* when compared to *C. populeti* and *E. tiliae*. This preference for *M. caryae* was also recorded when the aphid and *A. jamatonica* were offered as prey. In spite of this, we did not find differences in the first prey attacked in any of the two cafeteria experiments indicating that the first choice was random, probably because starved ladybirds do not miss any chance to feed [23]. The preference for *M. caryae* suggests that the potential control effect of *O. conglobata* in urban areas that included the black walnut (specific host of *M. caryae*) could be biased towards this aphid. However, in the cafeteria experiment with the three aphids, the number of *C. populeti* consumed by adults and larvae of *O. conglobata* after 24 h was much lower than those reported by [16].

Prey acceptability by a predator is the first step of a feeding process leading to its development and reproduction. However, a prey may be accepted and eaten repeatedly but may not contribute to that objective [24,25,26]. Therefore, acceptability is not really a measure of prey suitability and the results of the present study should be taken as preliminary information about *O. conglobata* as a biocontrol agent. More detailed experiments should be developed to determine the fitness and functional responses of this coccinellid feeding on different prey species in order to determine its suitability to be mass reared for biological control purposes.

## Figures and Tables

**Table 1:** Mean values ± s.e. of number of prey individuals consumed at 1, 2, 4, and 8 h after the release of adults and larvae of *C. conglobata* into the non-choice experimental arenas.

	Number of Individuals Consumed			
Prey	1 h	2 h	4 h	8 h
ADULTS				
*M. caryae*	10.7 ± 0,5 a *	13.0 ± 0,5 a	15.5 ± 0,5 a	18.1 ± 0,4 a
*C. populeti*	2.6 ± 0.3 bcd	4.8 ± 0.3 b	6.0 ± 0.4 c	7.3 ± 0.5 c
*E. tiliae*	4.0 ± 0.4 b	5.4 ± 0,4 b	7.2 ± 0.5 bc	9.3 ± 0.4 bc
*A. gossypii*	2.5 ± 0.4 cd	4.1 ± 0.4 b	6.5 ± 0.5 bc	9.2 ± 0.6 bc
*A. craccivora*	1.5 ± 02 d	2.3 ± 0.2 c	3.4 ± 0.2 d	4.7 ± 0.3 d
*A. nerii*	0.1 ± 0.1 e	0.2 ± 0.1 d	0.2 ± 0.1 e	0.6 ± 0.1 e
*R. padi*	3.2 ± 0.3 bc	5.5 ± 0.4 b	8.0 ± 0.5 b	11.3 ± 0.8 b
*A. jamatonica*	3.6 ± 0.4 bc	5.4 ± 0.5 b	8.0 ± 0.6 b	10.7 ± 0.7 bc
LARVAE				
*M. caryae*	6.0 ± 0.6 a	8.3 ± 0.7 a	10.5 ± 0.8 a	13.7 ± 0.6 a
*C. populeti*	1.6 ± 0.2 bc	2.2 ± 0.2 cd	2.9 ± 0.2 de	3.8 ± 0.2 de
*E. tiliae*	2.3 ± 0.3 b	3.3 ± 0.3 bc	4.4 ± 0.4 cd	6.5 ± 0.5 cd
*A. gossypii*	2.5 ± 0.2 b	3.6 ± 0.2 bc	5.1 ± 0.3 c	7.0 ± 0.5 c
*A. craccivora*	0.8 ± 0.1 cd	1.4 ± 0.2 de	2.0 ± 0.3 e	3.0 ± 0.3 e
*A. nerii*	0.0 ± 0.0 d	0.0 ± 0.0 e	0.1 ± 0.1 f	0.1 ± 0.1 f
*R. padi*	1.7 ± 0.2 bc	2.7 ± 0.2 cd	3.8 ± 0.3 cde	5.0 ± 0.4 d
*A. jamatonica*	2.7 ± 0.3 b	4.5 ± 0.3 b	7.2 ± 0.5 b	9.6 ± 0.5 b

* For each life stage and time interval, values of consumption followed by letters repeated in more than one prey mean the absence of significant differences between the consumption of these preys (Tukey’s test, *P* < 0.05).

**Table 2:** Rodgers’s index (mean ± s.e.) for adults, L3 nymphs of *O. conglobata* and both stages together when they were offered simultaneously with three aphid species (larvae *n* = 30, adults *n* = 30).

Prey	Adults	Larvae	Both Stages
*M. caryae*	1.00 ± 0.00 a *	1.00 ± 0.00 a	1.00 ± 0.00 a
*E. tiliae*	0.63 ± 0.02 b	0.61 ± 0.03 b	0.62 ± 0.02 b
*C. populeti*	0.59 ± 0.03 b	0.62 ± 0.03 b	0.61 ± 0.02 b

* Within each life stage values followed by different letters are significantly different (Tukey’s test, *P* < 0.05).

**Table 3:** Rodgers’s index (mean ± s.e.) for adults, L3 nymphs of *O. conglobata* and both life stages together when they were offered the aphid *M. caryae* and the psyllid *A. jamatonica* (larvae *n* = 31, adults *n* = 31) simultaneously.

Prey	Adults	Larva	Both Stages
*M. caryae*	1.00 ± 0.00 a *	0.98 ± 0.01 a	0.99 ± 0.01 a
*A. jamatonica*	0.65 ± 0.02 b	0.70 ± 0.03 b	0.67 ± 0.02 b

* Within each life stage values followed by different letters are significantly different (Tukey’s test, *P* < 0.05).

## References

[1] Iperti G. (1999). Biodiversity of predaceous coccinellidae in relation to bioindication and economic importance. Agric. Ecosyst. Environ..

[2] Lumbierres B., Starý P., Pons X. (2005). Parasitoids and predators of aphids associated with public green areas of Lleida (NE Iberian Peninsula). Adv. Hortic. Sci..

[3] Ameixa O.M.C.C., Honek A., Martinkova Z., Kindlmann P. (2010). Position of *Harmonia axyridis* in aphidophagous guilds in the Czech Republic. IOBC/WPRS Bull..

[4] Rondoni G., Onofri A., Ricci C. (2012). Laboratory studies on intraguild and cannibalism among coccinellid larvae (Coleoptera: Coccinellidae). Eur. J. Entomol..

[5] Yakhontov V., Hodek I., Junk W. (1966). Food specificity in Syrphidae and Coccinellidae of Central Asia. Proceedings of the Ecology of Aphidophaga.

[6] Wakgari M., Rai A.K. (2011). Effect of weather factors on *Busseola fusca* (Fuller) Lepidoptera: Noctuidae and its effect predator *Oenopia conglobata* (L.) (Coccinellidae) on sorghum in Ethiopia. Indian J. Entomol..

[7] Hodek I., Honěk A. (1966). Ecology of Coccinellidae.

[8] Mehrnejad M.R., Jalali M.A. (2004). Life history parameters of the coccinellids beetle *Oenopia conglobata contaminata*, an important predator of the common pistachio psylla, *Agonoscena pistaciae* (Hemiptera: Psylloidea). Biocontrol Sci. Technol..

[9] Pons X., Lumbierres B. Control integrado de plagas en espacios verdes urbanos. Proceedings of the 12th Symposium Sanidad Vegetal: Hacia la gestión integrada de plagas.

[10] Lumbierres B. (2016). La marieta rosa (*Oenopia conglobata*). Lignosa.

[11] Krebs C.J. (1999). Ecological Methodology.

[12] (2004). SAS/STAT User’s Guide.

[13] Blackman R.L., Eastop V.F. (2014). Aphids on the World’s Plants. An Online Identification and Information Guide. http://www.aphidsonworldsplants.info.

[14] Pons X., Comas J., Albajes R. (1995). Occurrence of holocyclic and anholocyclic populations of *Rhopalosiphum padi* and *Sitobion avenae* (Hom., Aphididae) in the northeast of Spain. J. Appl. Entomol..

[15] Pons X., Lumbierres B., Madeira F., Starý P. Aphid-parasitoid diversity in urban green areas: A background for conservative control strategies. Biodiversity.

[16] Sadeghi S.E., Mojib H.G.Z., Jalali J., Haji Z.J. (2004). Investigation on the biology of lady beetle *Oenopia conglobata* (L.) on poplar aphid *Chaitophorus leucomelas* (Koch) in laboratory conditions. Pajouhesh Sazandegi (Natural Resources).

[17] Mojib H.G.Z., Jalali S.J., Sedeghi S.E., Yousefpour M. (2009). Introduction of lady beetle *Oenopia conglobata* (L.) as predator of ulmus aphid *Tinocallis saltans* Nevski in Guilan province and biology of ladybeetle in laboratory conditions. Iran. J. Biol..

[18] Yaşar B., Özger S. (2005). Functional response of *Oenopia conglobata* (L.) (Coleoptera: Coccinellidae) on *Hyalopterus pruni* (Geoffroy) (Homoptera: Aphididae) in three different size arenas. Türk Entomol. Derg..

[19] Rondoni G., Ielo F., Ricci C., Conti E. (2017). Behavioural and physiological responses top rey-related cues reflect higher competitiveness of invasive vs. native ladybirds. Sci. Rep..

[20] Ninkovic V., Pettersson J. (2003). Searching behaviour of the sevenspotted ladybird, *Coccinella septempunctata*—Effects of plant-plant odour interaction. Oikos.

[21] Iperti G. (1966). Comportement naturel des Coccinelles aphidophages du Sud-Est de la France: Leur type de spécificité, leur action prédatrice sur *Aphis fabae* L.. Entomophaga.

[22] Blackman R.L. (1967). Selection of aphid prey by *Adalia bipunctata* L. and *Coccinella 7-punctata* L.. Ann. App. Biol..

[23] Nevded O., Salvucci S. (2008). Ladybird *Coccinella septempunctata* (Coleoptera: Coccinellidae) prefers toxic prey in laboratory choice experiment. Eur. J. Entomol..

[24] Obrycki J.J., Orr C.J. (1990). Suitability of three prey species for Neartic populations of *Coccinella septempunctata*, *Hippodamia variegata* and *Propylea quatuordecimpunctata* (Coleoptera: Coccinellidae). J. Econ. Entomol..

[25] Kalushkov P., Hodek I. (2004). The effects of thirteen species of aphids on some life history parameters of the ladybird *Coccinella septempunctata*. Biocontrol.

[26] Mignault M.P., Roy M., Brodeur J. (2006). Soybean aphid predators in Quebec and suitability of *Aphis glycines* as prey for three Coccinellidae. Biocontrol.

